# Tonsillitis and Sinusitis: A Narrative Review of Pathogenesis, Diagnosis, and Management

**DOI:** 10.7759/cureus.47192

**Published:** 2023-10-17

**Authors:** Harshit Singh, Asmi Bhatt, Mayank Kumar, Prasad Deshmukh

**Affiliations:** 1 Otolaryngology, Jawaharlal Nehru Medical College, Datta Meghe Institute of Higher Education and Research, Wardha, IND; 2 Medicine and Surgery, Jawaharlal Nehru Medical College, Datta Meghe Institute of Higher Education and Research, Wardha, IND; 3 Community Medicine, Jawaharlal Nehru Medical College, Datta Meghe Institute of Higher Education and Research, Wardha, IND

**Keywords:** upper respiratory tract infections, antibiotics, paranasal sinuses, streptococcus pyogenes, sinusitis, tonsillitis

## Abstract

The review aims for a comprehensive examination of tonsillitis and sinusitis, covering their pathophysiology, diagnosis, and management, with a focus on recent breakthroughs and therapeutic practices. Tonsillitis, marked by inflammation of the tonsils, can result from viral or bacterial infections, particularly *Streptococcus pyogenes*, with attention to antibiotic resistance trends. This review discusses clinical manifestations, diagnostic criteria, and the importance of distinguishing viral from bacterial causes. Therapeutic interventions like antibiotics and tonsillectomy indications are evaluated within evolving guidelines. Regarding sinusitis, it explores its origins, contributing factors, and classification based on duration and pathophysiology. Viral infections, allergens, and structural anomalies' roles in pathogenesis are highlighted. Diagnostic modalities like imaging and endoscopic exams are assessed for their efficacy in guiding management decisions. The importance of precise diagnosis through clinical examination, microbiological testing, and imaging is emphasized for informed treatment choices. This review also delves into minimally invasive surgical procedures, particularly endoscopic sinus surgery and tonsillectomy, showcasing progress in these areas. In summary, it provides insights into tonsillitis and sinusitis, offering perspectives on their aetiology, diagnosis, and treatment while integrating current research and clinical standards to enhance patient care and healthcare resource utilization.

## Introduction and background

Upper respiratory tract infections (URTIs) are a pervasive and burdensome health concern affecting individuals of all ages globally. Among the diverse URTIs, tonsillitis and sinusitis emerge as two distinct yet interconnected conditions that pose significant challenges to patients and healthcare systems. Tonsillitis, characterized by inflammation of the tonsils located at the back of the throat, and sinusitis, an inflammation of the paranasal sinuses within the facial bones, collectively contributed to a substantial portion of medical visits, antimicrobial prescriptions, and missed school or work days. This review article embarks on a comprehensive journey to elucidate the intricate facets of these common URTIs, aiming to foster a deeper understanding of their pathogenesis, clinical presentation, diagnostic intricacies, and evolving management strategies. The intricate network of tissues and structures comprising the upper respiratory tract is the frontline defence against airborne pathogens. The tonsils, small masses of lymphoid tissue located at the juncture of the oral cavity and pharynx, play a pivotal role in the immune response by detecting and neutralizing invading microorganisms. Similarly, the paranasal sinuses, interconnected hollow spaces lined with mucosa, contribute to the humidification and filtration of inhaled air while also playing a part in immune surveillance. Understanding the anatomical significance of these regions is vital in comprehending the susceptibility of the tonsils and sinuses to infections [1].

Tonsillitis encompasses a spectrum of infectious and inflammatory processes that can be bacterial or viral. *Streptococcus pyogenes*, commonly known as group A *Streptococcus *(GAS), emerges as a primary bacterial pathogen responsible for acute bacterial tonsillitis, presenting symptoms such as sore throat, fever, and cervical lymphadenopathy. While viral infections account for a significant proportion of tonsillitis cases, the potential for bacterial complications and the growing concern over antimicrobial resistance emphasize the need for accurate differentiation between bacterial and viral aetiologies. This challenge sets the stage for a nuanced discussion on the diagnostic approaches and treatment strategies central to effective patient management. On the other hand, sinusitis encompasses an array of inflammatory responses involving the paranasal sinuses. Various factors influence its pathogenesis, including viral infections, allergies, anatomical variations, and impaired mucociliary clearance. As inflammation occurs within the sinuses, patients often experience facial pain or pressure, nasal congestion, and mucopurulent nasal discharge. Sinusitis's chronicity and relapsing nature underscore the need for tailored diagnostic approaches to accurately identify contributing factors and guide appropriate therapeutic interventions [2].

Navigating the diagnostic landscape of tonsillitis and sinusitis is a formidable task, requiring an amalgamation of clinical understanding and technological advancements. Clinical evaluation, microbiological testing, and diagnostic imaging are cornerstones in determining these infections' aetiology and severity. Recent advances in diagnostic modalities, such as high-resolution imaging and molecular testing, have provided clinicians with valuable tools to enhance diagnostic accuracy and guide personalized treatment strategies.

In conclusion, tonsillitis and sinusitis are significant components of URTIs, with their intricate pathogenesis and clinical complexities warranting focused attention. This review article explores these conditions comprehensively, delving into their respective pathophysiological mechanisms, diagnostic challenges, and evolving management paradigms. By shedding light on the multifaceted nature of tonsillitis and sinusitis, this review seeks to optimize patient care, improve diagnostic accuracy, and mitigate the societal burden imposed by these familiar yet intricate URTIs [3].

## Review

Methodology

Literature Search

Various databases, such as PubMed, Cochrane Library, and Google Scholar, were utilized. Our search included specific keywords relevant to our study, including "streptococcal tonsillitis", "acute bacterial sinusitis", "microbiology", "treatment", "prevalence", and "burden of disease". We also manually searched the reference lists of relevant articles published until 2022 to identify additional studies.

Inclusion and Exclusion Criteria

Incorporated in our research were studies that explored the various aspects of sinusitis, such as microbiology, epidemiology, diagnosis, treatment, and prevention. However, we opted to exclude non-English studies and those that did not undergo the peer-review process.

Data Extraction and Synthesis

To conduct our analysis, we obtained information from each study that was included in the research. To evaluate this collected information effectively, we employed a narrative approach, which allowed us to provide an overview while highlighting important and current details.

Data Analysis

**Figure 1:**
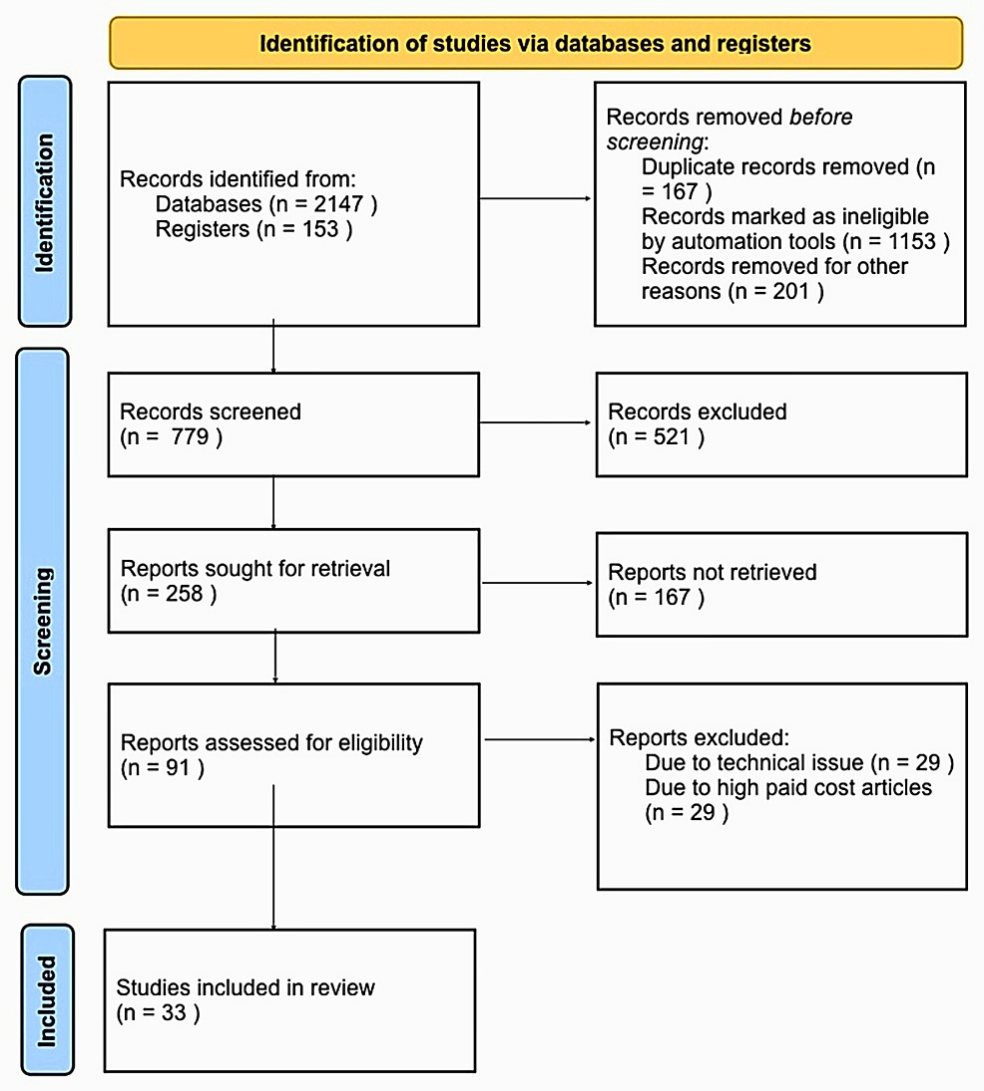
Search strategy utilized for the review.

In our analysis, we employed a qualitative method to examine the data. We focused on identifying recurring themes and patterns that emerged from the studies included in our research. Furthermore, we utilized descriptive statistics as a means of succinctly summarizing the findings obtained. The total number of articles that were screened was 91, out of which only 33 articles were utilized for this narrative review. Figure 1 shows the search strategy utilized for the review.

Acute bacterial sinusitis

Sinusitis is when the mucosal covering of one or more paranasal sinuses gets inflamed. It can be broken down into acute (lasting less than 30 days), subacute (lasting between 30 and 90 days), and chronic (lasting more than 90 days) diseases based on how long the symptoms last [1-4]. Acute sinusitis can be caused by several things, such as viral, bacterial, or fungal infections or exposure to environmental allergens and irritants [5]. A secondary bacterial illness in the sinuses usually causes acute bacterial sinusitis (ABS). According to studies, about 7.5% of URTIs in children are also accompanied by ABS [6-8]. Even though it happens a lot, ABS is often overlooked in young children because the signs are not obvious, and people think it is rare in this age group [9]. Without the proper medical care, ABS can lead to mild or chronic sinusitis, which can be very painful or even kill you. If a general care doctor does not know much about ABS, it might be hard for them to figure out what is wrong and how to treat it. The number of times or how often something happens is also important. By the time a child is three years old, it is thought that about 10% of them will have had at least one case of ABS [10]. Also, it is thought that complications due to ABS happen in 7.5% of URTIs. A study was done on 294 children between the ages of six and 35 months to determine how often they got upper respiratory tract illnesses. The study was done over a year, and 1295 cases of URTI were recorded. ABS was found to be a problem in 73 (out of the total group size of 100) of the 103 (or 8%) episodes that were seen in 73 children. In a separate observational cohort study with 236 children aged 48 to 96 months, 327 cases of URTIs with symptoms were reported over one year [11,12]. Out of the 327 cases of symptomatic URTI, 29 (8.8%) were found to be cases of sinusitis in 24 children. Children who go to daycare are two to three times more likely to get ABS after a viral URTI than children who do not go to daycare [13].

Etiopathogenesis

Sinusitis can be caused by a buildup of mucus in the sinuses that cannot drain because the ciliary apparatus is not working well, there are not enough of them, the sinus ostia are blocked, or the liquids are too thick or come out too much [2,7,14]. Most of the time, the presence of a URTI is what makes ABS happen. Upper respiratory illness (URTI) is the most common cause of mucosal oedema, which can block sinus openings. Mucosal oedema and occlusion of the ostia can also be caused by allergic rhinitis, cystic fibrosis, immune deficiency, facial injuries, diving, swimming, and using nasal decongestants too much. Enlarged adenoids, nasal polyps, foreign bodies, a deviated nasal septum, craniofacial abnormalities, and choanal atresia can cause sinus ostial blocking. Also, blocked sinuses can cause negative sinus pressure, which lowers the amount of oxygen in the sinus canal. This airdrop could draw mucus and germs from the lungs into the blocked sinus [6,7]. Still, the sinuses keep making fluids, which build up inside the sinuses. So, this buildup makes a good place for bacteria to grow [7].

Clinical Manifestations

There are three distinct manifestations associated with ABS. The prevailing manifestation typically involves enduring symptoms and indications of an upper respiratory infection (coughing, nasal congestion, and discharge) lasting more than 10 days with no recovery. Even though children who have an uncomplicated URTI may still experience symptoms by the 10th day, it is almost always observed that these symptoms have improved [2,3]. The cough is typically observed during the daytime but frequently exacerbates throughout the night [2,3]. The latter phenomenon could potentially arise due to irritation of the pharyngeal wall caused by postnasal drip when in a supine position. A cough that exclusively manifests during the night is more suggestive of postnasal drip or reactive airway disease. Accentuation of cough at night or during sleep should be studied in relation to the variation of the levels of adrenalin and cortisone during sleep. The cough tends to increase in intensity as time progresses. The prevalence of nasal congestion exceeds that of nasal discharge. The nasal discharge commonly presents in an anterior location, although it can also manifest in a posterior location. The discharge may exhibit variations in consistency, ranging from thin to thick, and can manifest as clear, serous, mucoid, mucopurulent, or purulent. The second presentation is characterized by a URTI with more pronounced symptoms, such as a high temperature of over 39°C and a purulent nasal discharge that is pigmented, thick, and opaque. These symptoms last at least three consecutive days, longer than the typical duration. In this matter, it is commonly observed that fever is typically not present in a straightforward URTI [15]. Fever, if present, typically manifests as a little elevation in body temperature, emerges at an early stage of the disease progression, and subsides within the initial 48 hours [15]. The presence of facial discomfort or periorbital oedema may be observed. Youngsters typically exhibit signs of illness [16].

The third presentation is characterized by biphasic or deteriorating symptoms, sometimes called "double sickness" [7,15]. The affected children experience early symptoms consistent with an uncomplicated viral URTI. Following a period of improvement, symptoms have significantly deteriorated, characterized by an aggravation of nasal congestion or discharge, daytime cough, or both [7,15]. A new fever may be observed or reoccur if it was already present at the beginning of the disease [7,15].

Diagnosis

Clinical evaluation and strict clinical criteria are the backbone of diagnosing acute sinusitis. These criteria include URTI symptoms that have persisted for more than 10 days without showing signs of resolution [17]. In addition, purulent nasal discharge in the early stages of a URTI that persists for at least three days is diagnostic of acute sinusitis. Additional diagnostic criteria [1] include the presence of biphasic symptoms or the worsening of symptoms (termed two-fold sickness). Endoscopic examination (rhinoscopy) for evaluation of the nasal mucosa and the areas where sinuses drain is an easy diagnostic and superior to imaging. It also helps to identify adenoid pathologies in children as well as confirm the presence of postnatal drip and its nature.

Differential Diagnosis

ABS should be distinguished from various other conditions, including a common URTI, acute viral sinusitis, pertussis, pneumonia, bronchiolitis, the presence of a foreign substance in the nasal cavity, infected adenoids, rhinitis medicamentosa, allergic rhinitis, and vasomotor rhinitis [2,18,19].

Treatment

The goals of treating children with ABS are to speed up their recovery and get them better as soon as possible, lessen the severity and length of their symptoms, eliminate the pathogens that are causing them, and stop any suppurative complications or chronic or recurrent sinusitis from happening [2,18,19]. Antibiotic treatment for ABS should start as soon as possible in children with serious initial symptoms, such as high fever and purulent nasal discharge, or a worsening course called "double sickening" [2,18,19]. The American Academy of Pediatrics (AAP) says this is the case. When managing children with ABS, doctors must decide whether to start antibiotic treatment immediately or wait three days to see if the symptoms improve on their own [20]. ABS leads to a fever, fluid from the nose, and a cough that lasts at least 10 days. In the second case, antibiotic treatment should be started if the child's state worsens or does not improve after three days of observation [2,18,19]. People have said that medicines like amoxicillin, amoxicillin-clavulanate, cefpodoxime, cefdinir, levofloxacin, ceftriaxone, cefpodoxime, cefuroxime, ampicillin-sulbactam, and ceftriaxone work well for children with ABS [2,18,19].

When choosing which antibiotic to use, there are many things to think about, such as the severity of the disease, the presence of risk factors, the likely causative organisms and their resistance patterns, how easy it is to give the dosage, how safe the drug is, and how much it costs [2,18,19]. The right amount of antibiotics should be taken at the right time. Most people now agree that antibiotic treatment should continue for at least seven more days after the patient stops having symptoms. Previous studies show that this may need a 10 to 21-day course of antibiotics. Patients should feel better if they start the right drug treatment within 72 hours [2,5,6]. The AAP says that amoxicillin or amoxicillin-clavulanate is the best way to treat ABS [2,18,19]. Current consensus says that amoxicillin-clavulanate should be the first choice for treating uncomplicated ABS in children. It should be given orally at a standard dose of 45 mg/kg/day of the amoxicillin component (with a maximum daily dose of 1.75 g) as long as there are no signs of antibacterial resistance [2,18,19].

Amoxicillin can also be given in two equal doses per day, each of which is 90 milligrams per kilogram of body weight (with a maximum of 4 grams per day). Patients with severe ABS or uncomplicated acute sinusitis who are at risk for serious sickness or antibiotic resistance are told to take high-dose oral amoxicillin-clavulanate (90 mg/kg/day of the amoxicillin component, split into two doses; maximum of 4 g/day). Many things can lead to germs becoming resistant. Children younger than two years old, people who have recently been in the hospital, people who go to daycare, people who have been deimmunized or only partially immunized with pneumococcal conjugate vaccine, and people who do not have enough immune cells are more likely to get pneumococcal disease [2,18,19]. There are some alternatives to high-dose amoxicillin-clavulanate, such as cefpodoxime, cefdinir, and levofloxacin. Cefpodoxime can be taken orally between 10 mg/kg/day and 400 mg/day, which can be split into two amounts. The suggested dose of cefdinir taken by mouth is 14 mg/kg/day, with a maximum daily dose of 600 mg. This amount can be given all at once or split in half and given in two equal doses. Levofloxacin should be taken by mouth at 10-20 mg/kg/day, with a maximum daily dose of 500 mg. This can be given all at once or in two doses that are spaced out evenly. Children with emesis who cannot take pills by mouth can get ceftriaxone through an IV or muscle injection once a day at 50 mg per kilogram (with a maximum dose of 1 g per day). Once the cause of the vomiting has been fixed, it is best to give the drug by mouth [2,18,19]. Children with acute allergic hypersensitivity to penicillin should take 10-20 mg/kg/day of levofloxacin by mouth. A single dose and two doses at different times are fine [2,15,19]. Systemic levofloxacin could cause damage to the muscles and bones, so it should be used with care when treating kids. However, since there are not any other safe and successful ways to treat children, systemic levofloxacin should be used. With the addition of doxycycline, it might be easier to treat older kids allergic to lactam medicines. Children with a minor delayed hypersensitivity reaction to penicillin can be treated with oral cefpodoxime at a dose of 10 mg/kg/day (with a maximum daily dose of 400 mg) split into two doses or oral cefdinir at a dose of 14 mg/kg/day (with a maximum daily dose of 600 mg) given as a single dose or split into two doses [2,18,19].

Prognosis

Uncomplicated ABS generally exhibits a favourable response to suitable antibiotic treatment, leading to noticeable clinical improvement within 72 hours. The condition above does not independently result in notable death rates. Complex abdominal compartment syndrome (ACS) can potentially result in adverse health outcomes, including morbidity and, in rare cases, fatality [2,18,19]. The occurrence of ABS is infrequent among children who are in good health. Nevertheless, individuals who suffer from immunodeficiency, cystic fibrosis, nasal polyps, and immotile cilia syndrome are susceptible to experiencing recurrent ABS [6].

Streptococcal tonsillitis

A significant number of visits (over 12 million) are documented annually in the United States due to pharyngitis, making it a common illness seen in paediatric patients. Most pharyngitis occurrences requiring antibiotic treatment are caused by* Streptococcus pyogenes*, also known as GAS. Streptococcal pharyngitis can be prevented, or at least the severity of any following rheumatic fever, by using antibiotics to clear up any lingering germs in the throat. The incubation period for GAS is normally between two and five days, and it is spread between people by respiratory droplets. Since kids spend the most time indoors for school and extracurricular activities in late winter and early spring, this is also when GAS pharyngitis is most common. During the colder months, colonization is at its peak. GAS colonization of the throat is common among school-aged children, affecting about 20%. However, there is no proof that colonization contributes to disease transmission at present. The risk of a pharyngitis outbreak increases in crowded environments like schools and low- and middle-income countries. Pharyngitis caused by GAS* *can affect people of any age, but it most commonly appears in school-aged children, especially between the ages of seven and eight years.

Clinical Presentation

Without viral respiratory symptoms, GAS pharyngitis usually shows up as a rapid fever, sore throat, and inflamed tonsils that can be seen during an exam [21]. A popular type of lymphadenitis is in the front of the neck, which can be painful. Some other signs that may appear are a strawberry tongue, a red, enlarged uvula, and palatal petechiae. But it is important to understand that none of these symptoms is a sure sign of GAS pharyngitis and that they can also be signs of viral pharyngitis or Kawasaki disease. Aside from the ones that have already been stated, other clinical signs include stomach pain, nausea, and vomiting. Streptococcal testing should not be done on children who have signs of a viral disease, such as a fever, sore throat, cough, runny or stuffy nose, hoarse voice, diarrhoea, conjunctivitis, coryza, or oral ulcers [22,23]. The new results from Shapiro et al. back up the idea. Researchers found that kids with viral symptoms, especially rhinorrhea, were less likely to test positive for streptococcal antigen. Also, the chances of getting a good result from an antigen test decreased as the number of viral features increased. GAS* *tonsillitis in children younger than three years old rarely has the usual clinical symptoms. Subacute GAS infection usually affects young children and causes a slight fever, irritability, loss of appetite, stuffy nose, mucus and pus coming out of the nose, and swollen lymph nodes in the front [24-26].

Peritonsillar Abscess

A peritonsillar abscess (PTA) refers to the accumulation of purulence in the space between the tonsillar capsule and the pharyngeal constrictor muscle. PTA has clinical manifestations reminiscent of streptococcal pharyngitis. However, PTA may also accompany additional clinical symptoms such as dysphagia, odynophagia, drooling, a muffled voice, and trismus. During an examination, it is possible to observe a visibly swollen tonsil that displaces the uvula away from the abscess [27]. It has been shown that younger children may exhibit a lower likelihood of experiencing symptoms such as a sore throat. Instead, they may exhibit neck swelling and soreness during a medical examination [28].

Suppurative and Non-suppurative Sequelae

Children not accurately diagnosed with GAS tonsillitis are more likely to develop suppurative sequelae, such as otitis media, sinusitis, and PTA. Systematic studies have demonstrated that therapy can help reduce this risk [29]. Nevertheless, the potential consequences of not receiving therapy seem to be minimal. The study conducted by Little et al. revealed that the number of cases of GAS tonsillitis that needed to be treated to prevent one complication of otitis media or sinusitis was 193 for patients who were prescribed antibiotics during their initial visit and 174 for those who received a delayed antibiotic prescription [30]. Poststreptococcal acute glomerulonephritis is a less common consequence of group streptococcal tonsillitis than otitis media and sinusitis. Its yearly occurrence rate is 9.4 per 100,000 children under 20 years. The prevalence of this phenomenon is highest among individuals in the adolescent stage, particularly at the age of 13 years, as indicated by previous research [31]. In line with otitis media and sinusitis, recent observational evidence indicates that the administration of antibiotics immediately may not offer superior preventive benefits compared to delayed or no antibiotics in the context of PTA. This shows that early signs of a PTA may be detectable during the initial clinical presentation, as supported by the studies mentioned earlier [31,32]. The incidence of adverse effects resulting from antibiotic exposure has been determined to be significantly greater than the incidence of otitis media, sinusitis, and PTA that may occur as a consequence of untreated GAS pharyngitis [33]. According to a recent Cochrane review, administering antibiotics to children was associated with a heightened likelihood of experiencing adverse effects such as vomiting, diarrhoea, or rash.

Acute rheumatic fever (ARF) predominantly manifests in untreated school-aged children between the ages of five and 14 years with a previous history of untreated tonsillitis [21]. Rare instances of acute renal failure have been observed in young children between the ages of two and three years within high-risk groups [22]. The Jones criteria for the diagnosis of ARF underwent revision in 2015 to improve the ability to differentiate individuals with a low risk of ARF from those with a moderate or high risk [21]. This modification upholds both primary and secondary criteria; however, they vary for groups with low risk and those with moderate to high risk. The modifications made to the diagnostic criteria were a significant revision, considering that ARF is infrequently observed in developed countries and is no longer classified as a notifiable condition by the Centers for Disease Control and Prevention (CDC) [21,23,24]. Nonetheless, it is worth noting that ARF and rheumatic heart disease remain significant contributors to illness in low- and middle-income nations and specific demographics. This underscores the criticality of promptly diagnosing and treating GAS tonsillitis in these groups, as it can greatly enhance the overall health of the population [21,23,24].

Diagnosis

The precise identification ofGAS pharyngitis is of utmost significance to mitigate the potential consequences of untreated infection and restrict the disease's spread. Given that the predominant cause of sore throats in paediatric patients is viral, it is imperative to ensure precise identification of the underlying cause of pharyngitis to avoid the unwarranted administration of antibiotics. Even when aggregated into prediction rules, clinical signs and symptoms lack reliability in diagnosing GAS pharyngitis in children [21,23,24]. The Infectious Diseases Society of America (IDSA) recommends the use of the rapid antigen detection test (RADT) as a definitive diagnostic method for detecting GAS pharyngitis. The sensitivity of RADT is commonly reported to range from 70% to 90% compared to blood agar plate culture, with specimen quality being a determining factor [21,23,24]. The IDSA and the American Heart Association advocate for using backup cultures in children who receive negative results from RADT. Conversely, the European guideline asserts that throat culture is not required following a negative RADT in children and adults presenting with an acute sore throat [21,23,24]. In children who have recently received treatment for GAS pharyngitis or when the incubation period of the RADT exceeds the recommendations provided by the manufacturer, there is a possibility of obtaining false-positive results [21,23,24].

Treatment

Patients presenting with clinical manifestations of pharyngitis and laboratory evidence confirming GAS as the underlying pathogen should undergo antibiotic therapy, as outlined in Table 1. To date, there is no reported evidence of GAS resistance to penicillin. Penicillin is considered the preferred treatment for GAS pharyngitis because of its specific antimicrobial activity, affordability, and effectiveness in preventing ARF. Amoxicillin exhibits superior palatability as a suspension compared to penicillin V. It demonstrates equivalent efficacy when administered once daily (50 mg/kg, maximum 1000 mg) over 10 days. In cases where medication adherence is doubtful, penicillin G-benzathine may be administered via intramuscular (IM) injection as a single dosage. It is important to note that this mode of administration may cause discomfort or pain. For individuals with a non-anaphylactic allergy to penicillin, it is considered appropriate to administer a first-generation cephalosporin, such as cephalexin, as a therapeutic option. In individuals exhibiting anaphylaxis or type 1 hypersensitivity reactions to penicillin, administering either clindamycin or a macrolide antibiotic, such as azithromycin, may be a suitable therapy option. In the case of children who encounter a relapse of laboratory-confirmed GAS pharyngitis immediately after concluding a course of antibiotic treatment, it is recommended to administer either the same antibiotic or an alternate option. Such alternatives may include a narrow-spectrum cephalosporin, amoxicillin-clavulanate, or a macrolide. It is advisable to do laboratory confirmation of GAS isolate susceptibility when non-beta lactam drugs are employed, as certain regions have reported significant rates of resistance [21,23,24]. Using sulfonamides, tetracyclines, and fluoroquinolones is contraindicated for managing GAS pharyngitis.

**Table 1:** Antibiotic options for treatment of GAS pharyngitis. GAS: group A *Streptococcus*; IM: intramuscular; U: unit; mg: milligram; kg: kilogram.

Initial and recurrent episodes	Dose and duration	Considerations
Penicillin V (oral)	If 27 kg: 250 mg per dose, two to three times daily for 10 days. If > 27 kg: 500 mg per dose, two to three times daily for 10 days	Preferred therapy
Amoxicillin	50 mg/kg once daily for 10 days; max dose 1000 mg	Preferred therapy
Penicillin G benzathine (IM)	If < 27 kg: 600,000 U (375 mg) as a single dose. If 27 kg: 1.2 million U (750 mg) intramuscular as a single dose	Preferred therapy in situations of poor medication adherence

The presence of multiple positive tests forGAS pharyngitis could indicate the persistent colonization of the throat by GAS in cases of viral pharyngitis or pharyngitis caused by other factors. Antibiotic therapy to eliminate GAS pharyngeal carriage is not typically recommended due to the low likelihood of carriers experiencing difficulties and the minimal danger of transmitting the infection. The eradication of GAS pharyngeal carriage may be warranted in specific situations, including instances of outbreaks of GAS pharyngitis in enclosed or partially enclosed communities, community-wide outbreaks of invasive GAS disease, or complications associated with GAS, such as ARF.

Repeated occurrences of GAS pharyngitis within a household over an extended period, despite receiving appropriate antibiotic treatment are noted. The efficacy of penicillin or amoxicillin in eliminating GAS pharyngeal carriage may be comparatively lower than that of alternative antibiotic treatments. Reacquisition may occur after successfully eradicating a condition, and the persistence of pharyngeal carriage might extend for several months to several years. The regular recommendation does not support using tonsillectomy as a standalone measure to reduce the frequency of episodes of GAS pharyngitis. Tonsillectomy should be considered if the frequency of pharyngitis episodes meets the following criteria: at least seven episodes within a single year, five episodes per year for two years, and three episodes per year for two years.

Performing a tonsillectomy may be considered in cases of recurrent pharyngitis, particularly when additional criteria are present, such as a history of PTA or numerous sensitivities to antibiotics.

## Conclusions

Physicians frequently encounter the issue of distinguishing between acute viral upper respiratory infection (URI) and ABS due to the overlapping clinical characteristics of these diseases. In general, ABS is characterized by enduring symptoms and indications of a URI that remain for 10 days with little amelioration. ABS may manifest as a URI accompanied by an elevated body temperature and the production of purulent nasal discharge that persists for a minimum of three consecutive days. Additionally, symptoms of ABS may exhibit a biphasic pattern or demonstrate progressive deterioration. The identification of ABS primarily relies on clinical assessment. Imaging modalities for assessing paranasal sinuses are not advised as a primary diagnostic tool for ABS unless there is a suspicion of associated problems. The prevailing agreement among experts is that amoxicillin-clavulanate, administered at a normal dosage of 45 mg/kg/day of the amoxicillin component, is considered the primary treatment for uncomplicated ABS in cases where there is no suspicion of antibacterial resistance. High-dose amoxicillin-clavulanate is the optimum treatment option for individuals with severe ABS or who risk developing severe disease or antibiotic resistance. There is an optimistic outlook for future high-quality, prospective clinical trials to yield additional insights into diagnosing and managing ABS in paediatric patients. These studies are expected to enhance specific recommendations for addressing this clinical condition. Tonsillitis is a frequently encountered condition that often necessitates medical intervention. Identifying GAS as the causative agent of tonsillitis is a prevalent concern among patients and parents. Viruses cause most instances of tonsillitis, and it is recommended to select which patients should undergo testing based on a thorough examination of their medical history, analysis of seasonal patterns of the disease, and consideration of physical examination results. This strategy reduces unnecessary testing and consequent antibiotic exposure, mitigating patient and societal healthcare expenditures.
